# Naturalistic visualization of reaching movements using head-mounted displays improves movement quality compared to conventional computer screens and proves high usability

**DOI:** 10.1186/s12984-022-01101-8

**Published:** 2022-12-09

**Authors:** Nicolas Wenk, Karin A. Buetler, Joaquin Penalver-Andres, René M. Müri, Laura Marchal-Crespo

**Affiliations:** 1 Motor Learning and Neurorehabilitation Laboratory, ARTORG Center for Biomedical Engineering Research, University of Bern, Freiburgstrasse 3, 3010 Bern, Switzerland; 2 Gerontechnology and Rehabilitation, ARTORG Center for Biomedical Engineering Research, University of Bern, Bern, Switzerland; 3 Department of Neurology, University Neurorehabilitation, University Hospital Bern (Inselspital), University of Bern, Bern, Switzerland; 4 Department of Cognitive Robotics, Delft University of Technology, Mekelweg 2, 2628 CD, Delft, The Netherlands

**Keywords:** Virtual reality, Augmented reality, Head-mounted display, Neurorehabilitation, Movement quality, Cognitive load, Motivation, Usability, Stroke

## Abstract

**Background:**

The relearning of movements after brain injury can be optimized by providing intensive, meaningful, and motivating training using virtual reality (VR). However, most current solutions use two-dimensional (2D) screens, where patients interact via symbolic representations of their limbs (e.g., a cursor). These 2D screens lack depth cues, potentially deteriorating movement quality and increasing cognitive load. Head-mounted displays (HMDs) have great potential to provide naturalistic movement visualization by incorporating improved depth cues, reduce visuospatial transformations by rendering movements in the space where they are performed, and preserve eye-hand coordination by showing an avatar—with immersive VR (IVR)—or the user’s real body—with augmented reality (AR). However, elderly populations might not find these novel technologies usable, hampering potential motor and cognitive benefits.

**Methods:**

We compared movement quality, cognitive load, motivation, and system usability in twenty elderly participants (>59 years old) while performing a dual motor-cognitive task with different visualization technologies: IVR HMD, AR HMD, and a 2D screen. We evaluated participants’ self-reported cognitive load, motivation, and usability using questionnaires. We also conducted a pilot study with five brain-injured patients comparing the visualization technologies while using an assistive device.

**Results:**

Elderly participants performed straighter, shorter duration, and smoother movements when the task was visualized with the HMDs than screen. The IVR HMD led to shorter duration movements than AR. Movement onsets were shorter with IVR than AR, and shorter for both HMDs than the screen, potentially indicating facilitated reaction times due to reduced cognitive load. No differences were found in the questionnaires regarding cognitive load, motivation, or usability between technologies in elderly participants. Both HMDs proved high usability in our small sample of patients.

**Conclusions:**

HMDs are a promising technology to be incorporated into neurorehabilitation, as their more naturalistic movement visualization improves movement quality compared to conventional screens. HMDs demonstrate high usability, without decreasing participants’ motivation, and might potentially lower cognitive load. Our preliminary clinical results suggest that brain-injured patients may especially benefit from more immersive technologies. However, larger patient samples are needed to draw stronger conclusions.**

## Introduction

Stroke is one of the most important sources of permanent disability worldwide with over 12 million new cases every year worldwide [1]. Stroke is defined as a “*disturbance of cerebral function, lasting more than 24 hours or leading to death, with no apparent cause other than of vascular origin*” [2]. Similar to other neurological dysfunctions—e.g., Parkinson’s disease, traumatic brain injury—, stroke survivors usually suffer from motor impairments such as muscle weakness, reduced movement workspace, and loss of movement quality, which limit their ability to perform activities of daily living (ADL) independently. Importantly, 21–44 % of stroke survivors also suffer from cognitive impairments that impact among others, memory, language, orientation, attention, and/or executive function [3].

When the potential for recovery remains—e.g., when there is no substantial damage to the corticospinal tract [4, 5]—relearning of movements after a brain injury can be achieved by enrolling into neurorehabilitation interventions. Neurorehabilitation aims to enhance patients’ functional movements during ADLs [6] and is accepted to be a form of motor (re)learning [7]. Neurorehabilitation can be optimized by promoting intensive [8] and task-specific [9] movement training that provides functional multi-sensory input to the central nervous system [10], known to lead to synaptic plasticity in the brain [11].

Robotic devices, together with virtual reality (VR) games, can provide intensive training in a motivating virtual environment (VE). Moreover, VR allows patients to visualize their movements in a VE and can provide meaningful goal/task-oriented exercises—e.g., realistic simulations of the ADLs to retrain—that can be adapted to the patients’ specific needs. Importantly, the use of VR has been shown to increase patients’ motivation—a factor known to enhance functional recovery [12–14] and facilitate motor learning through the release of dopamine known to support memory consolidation and neuroplasticity [15]. A recent meta-analysis of randomized clinical trials concluded that VR is, indeed, a promising technology for upper limb motor rehabilitation in post-stroke patients [16].

However, during conventional robotic VR-based neurorehabilitation, the VE is usually displayed on a two-dimensional (2D) surface (e.g., 2D screen) and patients interact with the VE via a symbolic virtual representation of their limbs (e.g., a cursor). Although this provides useful visual feedback, 2D screens draw patients’ attention away from their limbs, breaking the eye-hand coordination. This eye-hand coordination is known to aid goal-oriented movements, which might be already affected in brain-injured patients [17]. Furthermore, the reduced depth cues in 2D screens that do not provide stereo vision and the visuospatial transformation between the visualized movements space and the physical movement space might add an extra cognitive load to the patients [18, 19]. This could lead to a two-step learning phase, where patients first learn the visuospatial transformation before being able to focus on learning the physiological movements, wasting valuable rehabilitation time, as observed during robotic training with 2D screens [20]. Finally, most of the tasks used to evaluate the benefit of VR training on motor learning and transfer are rather simple to be easily controlled (e.g., reaching on a plane), yet those deviate from the movements usually performed in ADLs (e.g., reaching in the 3D space) [6].

Low-cost off-the-shelf Head-Mounted Displays (HMD) are now widely available, offering the possibility to provide a realistic virtual representation of the patient’s own limbs (avatar) in immersive VR (IVR) or the possibility of projecting virtual elements while still visualizing the patient’s own limbs with augmented reality (AR). The use of different VR displays might result in different motor performance compared to real movements [21]. For example, reaching movements performed towards targets located in the vertical plane have been shown to be slower, shorter, less straight, and less accurate when visualized on a 2D screen than movements performed in real life [19], while when using HMDs, movements seemed closer to the ones performed towards targets in the real life [22]. In a previous experiment with healthy young participants, we found that IVR HMD is associated with better movement quality in a 3D reaching task than 2D screens, especially when moving across several dimensions (horizontal, vertical, and in depth) [23]. Different visualization technologies could also have a different effect on participants’ cognitive load and psychological affects, e.g., motivation and usability. Indeed, in our previous experiment, we observed that healthy young participants’ motivation and system usability were higher with the IVR HMD compared to the 2D screen [24]. However, the visualization technology did not significantly impact their cognitive load, neither when measured with a parallel cognitive task [23], nor with subjective reports [24].

Thus, IVR and AR HMDs might offer benefits over 2D screens. First, their improved depth cues (over 2D screens) allow higher **movement quality** [23, 25]. Second, by displaying the movement in the same space where it is performed, the visuospatial transformation is reduced, potentially lowering the patients’ **cognitive load** [18]. Third, by using an animated avatar, the eye-hand coordination [17] could be preserved [18]. This more naturalistic interaction could potentially improve the system **usability**, ultimately increasing the inclusion and adherence of patients into VR-based interventions [24]. The patient’s **motivation** might also increase, either directly due to this more naturalistic interaction, or indirectly due to an increased perceived competence elicited by the improved movement quality. However, although HMDs are slowly entering the rehabilitation context, there is currently little understanding of their impact on neurorehabilitation [16, 26, 27].

Brain injuries are more preeminent at older ages. Furthermore, cognitive decline is associated with participants’ old age that might limit the usability of 2D screens in motor training [28, 29]. Therefore, in this study, we aimed at reproducing our previous study [23, 24] with two different populations, namely, with 20 healthy elderly participants (>59 y.o.; **Experiment 1**) and a small group of five acute brain-injured patients (**Experiment 2**). To facilitate the experiment with brain-injured patients, who suffered from motor impairments, we interfaced our VR/AR setup with a weight-support rehabilitation device (Armeo$\circledR$ Spring, Hocoma, Switzerland).

Based on previous results obtained in healthy young participants [18, 23–25], we formulated the following hypotheses: (1) IVR HMD would elicit better movement quality, less cognitive load, higher motivation, and a higher usability compared to the 2D screen; (2) With the 2D screen, we also expected that the movement quality would worsen when the reaching movement requires moving in the depth dimension at the same time than in another dimension (vertical and/or horizontal) compared to movements that do not require using the depth direction. We expected to see differences between visualization technologies in the cognitive load of elderly participants and brain-injured patients, as they are less cognitively fit than the healthy young participants from our previous experiment. Since we did not find conclusive results for the AR HMD in our previous study in terms of movement quality, cognitive load, motivation, and usability, we did not formulate hypotheses for this specific visualization technology, but kept it in the protocol to gather insights about the use of AR within aging and brain-injured populations.

## Methods

### Experiment 1—healthy elderly participants

#### Participants

Twenty participants without known motor or cognitive disorders aged from 60 to 89 years (74.22 ± 8.11) and without severe visual impairment (as indicated by themselves during the recruitment process when asked about the presence of uncorrected visual impairments by the experimenter) provided written informed consent to participate in this first experiment. Fourteen participants were strongly right-handed, one was mixed right-handed, and one was mixed left-handed [30], based on the “Edinburgh Handedness Inventory” [31]. Other demographic data is available in Table 1. Participants were recruited via word-of-mouth. The study was approved by the local Ethics Committee (ref.: 2017-02,195) and conducted in compliance with the Declaration of Helsinki. Participants did not receive any compensation for their participation in the study.

**Table 1:** Elderly participants’ demographic data

#	Gender	Age	Highest educational achievement	Experience with VR	Experience with video games	Hours spent playing video games per week in the last month
1	Male	84	Apprenticeship	1	2	3
2	Female	82	Apprenticeship	1	1	0
3	Female	68	High school	1	1	0
4	Female	72	Apprenticeship	1	1	0
5	Male	64	University or equivalent	2	1	0
6	Female	64	University or equivalent	1	1	0
7	Female	70	Apprenticeship	1	1	0
8	Male	60	University or equivalent	1	2	0
9	Male	67	Apprenticeship	1	1	0
10	Male	64	High school	1	2	3
11	Female	89	Apprenticeship	1	1	0
12	Female	71	Apprenticeship	4	1	0
13	Male	85	Apprenticeship	1	1	0
14	Male	84	University or equivalent	2	2	0
15	Male	76	High school	5	5	0
16	Female	78	University or equivalent	1	1	0
17	Male	73	University or equivalent	2	3	0
18	Male	77	University or equivalent	1	1	0
19	Male	72	Apprenticeship	5	5	0.5
20	Male	75	Apprenticeship	4	6	2

Experience with VR and video gaming rated from 1 (“Not at all”) to 7 (“Very much”)

#### Experimental setup

**Fig. 1:**
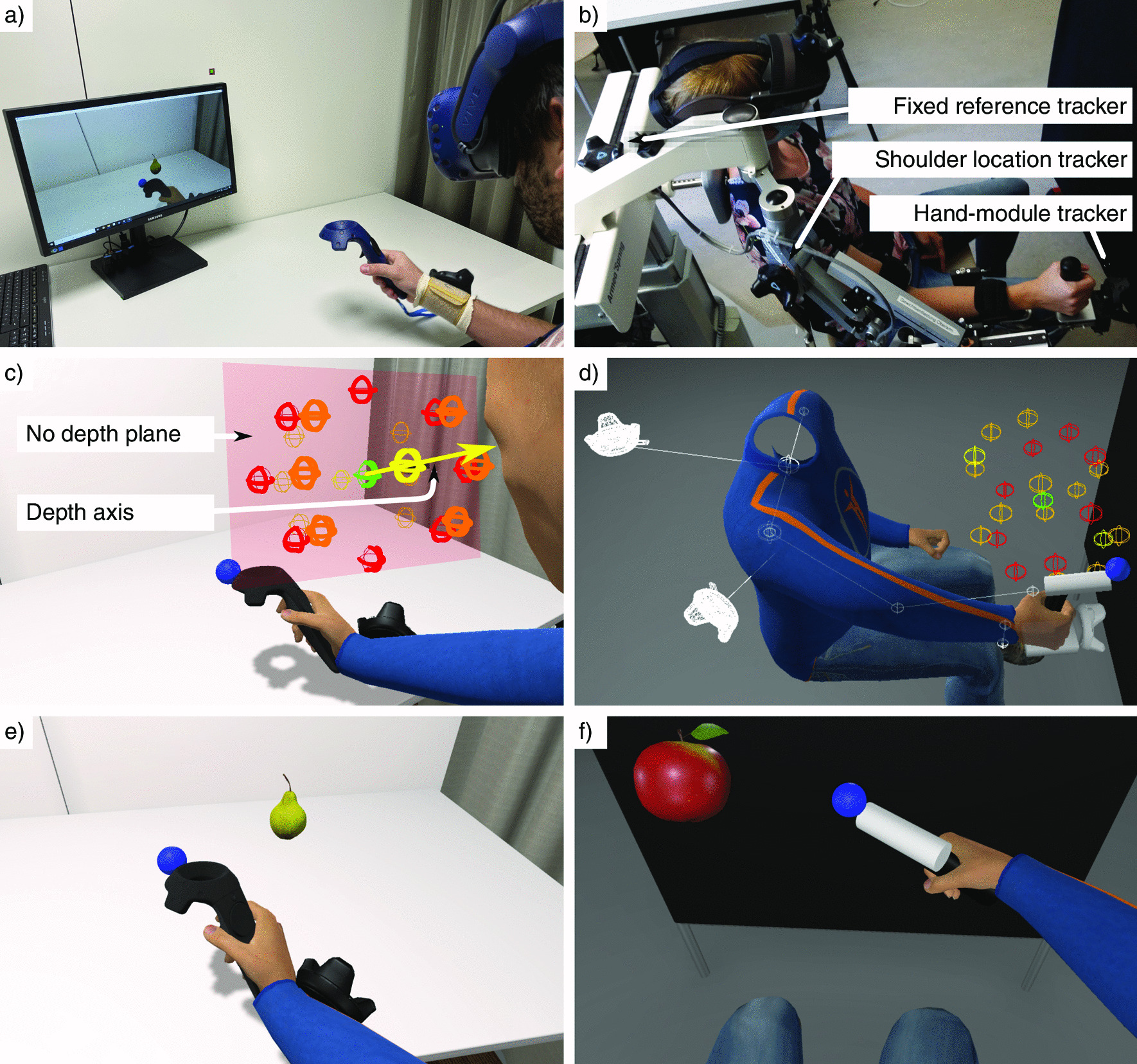
Experimental setups and Virtual environments. **a**, **c**, and **e**: Experiment 1; **b**, **d**, and **f**: Experiment 2; **a** and **b**: Experimental setup; **c** and **d**: Virtual room with the avatar, fruit locations color-coded by depth usage (red: no, yellow: only, orange: combined), the workspace center (green), and trackers plus arm animation hints; **e** and **f**: Participants’ view with the avatar from a first-person perspective and task elements (fruits and blue sphere)

The experiment was performed in a room with only artificial and controllable lighting (Fig. 1a). The participants sat on a lockable-wheeled chair set at a predefined fixed location in the room.

The IVR HMD used in the experimental setup (Fig. 1a) was an HTC Vive Pro (HTC, Taiwan & Valve, USA), tracked with two SteamVR$^{\textrm{TM}}$ Base Station 2.0. The IVR HMD was equipped with a 2880 × 1600 pixels Dual AMOLED 3.5” display with 90 Hz refresh rate and 110$^{\circ }$ field of view (diagonal). Participants wore three *HTC Vive trackers (2018)* attached to their right arms and shoulders, while holding an *HTC Vive controller (2018)* in their right hand (HTC, Taiwan & Valve, USA). We calibrated the IVR HMD by measuring the participants’ interpupillary distance and setting it with the dedicated wheel.

The AR HMD used was a Meta 2 (Meta Company, USA), with its “simultaneous localization and mapping” (SLAM) function disabled. The AR HMD was equipped with a 2560 x 1440 pixels display with 60 Hz refresh rate and 90$^{\circ }$ field of view (diagonal). The head tracking for AR was performed using an *HTC Vive tracker (2018)* fixated on the HMD to prevent that differences in tracking performance would affect our experiment results. To calibrate the AR HMD, participants were guided by the experimenter through the Meta 2 eye calibration software ($\sim$ 5 min).

The 2D computer screen used was a Samsung S24E560 (Samsung, South Korea) with a diagonal of 24 inches (with a resolution of 1920 × 1080 pixels, 60 Hz refresh rate and 60$^{\circ }$ field of view) and located on a table at an approximate distance of 1 m from the participant’s body. To align the tracking reference system to the participants for the 2D screen modality, we needed to know the participant’s initial head position and orientation. Therefore, participants wore the Meta 2 HMD tracked with the *HTC Vive tracker (2018)* in a first initialization phase. The experimenter quickly removed the HMD after calibration.

We employed a computer to run the VE and the experimental protocol with Windows 10 Home 64 bit edition (Microsoft, USA), 32 GB of DDR3 working memory, Intel Core i7-8700K (Intel Corporation, USA), and an NVIDIA GeForce GTX 1080 Ti (NVIDIA Corporation, USA).

#### Reaching and cognitive tasks

Participants were requested to perform a motor reaching task and, in parallel, a cognitive counting task. In the motor task, participants had to reach for and touch fruits (oranges, apples, and pears) that appeared at 22 pre-randomized locations within a pre-defined workspace of dimensions 35.88 × 28.47 × 37.26 cm (width × height × depth). The workspace was defined along three perpendicular axes: horizontal, vertical, and depth. The horizontal and vertical axes matched the horizontal and vertical screen coordinates, which rendered the VE from the avatar’s head location when looking at the center of the screen (origin of the VE Cartesian coordinate system). The workspace center was located at 19.8 cm on the right and at 44.4 cm in front of the transverse plane and 19.98 cm under the participants’ eyes on the longitudinal axis.

Two potential fruit locations within the workspace used only the depth axis (one on each side of the workspace center; Fig. 1c, locations in yellow). Eight potential locations did not use the depth at all (two using only the horizontal axis, two using only the vertical one, and four combining the horizontal and vertical axes; Fig. 1c, locations in red). Finally, twelve potential locations included the depth axis combined with at least another one (eight used combinations of the three axes and four combined the depth with the horizontal axis; Fig. 1c, locations in orange).

Only one fruit was visible at a time. To touch a fruit, participants had to reach towards it and “touch” it with a virtual blue sphere attached to the controller. As soon as the fruit was “touched”, it disappeared and a green sphere appeared in the center of the workspace. Participants were asked to then touch the green sphere (with the blue one) and to remain in contact with it until it disappeared and the next fruit appeared (unless the trial was over). The participants had to touch a total of 102 fruits, moving from the initial position marked by the green sphere, which were grouped into eight blocks of 6, 12, 12, 12, 18, 18, 18, and 6 fruits each. The green sphere where participants should move back after touching a fruit was visible, while remaining in contact with, for a random time interval between 0.4 and 0.6 s. This random dwelling time was selected to avoid that participants would anticipate the appearance of a new fruit, and thus, allowed us to detect more precisely the time between a new fruit appeared and the initiation of the movement (movement onset). The green sphere also appeared when starting a new block.

The blue and green spheres had a diameter of 4 cm and, when the blue sphere was in contact with the green one, the size of the green sphere increased by 10 % to offer visual feedback and increase tolerance to small hand displacements. To detect the contact between fruits and the blue sphere, the orange and apples had spherical colliders mapped to their shape with diameters of 10 cm and 7.52 cm, respectively. The lower part of the pear was mapped by a spherical collider with a diameter of 5.78 cm and the upper part with a capsule-like collider with a height of 7.31 cm and a diameter of 2.38 cm. All these dimensions were chosen so the whole workspace would fit within the visualization technology with the smallest field of view, i.e., the Meta 2 with a diagonal field of view of 90$^{\circ }$.

The participants performed a cognitive task in parallel to the motor task. They were asked to count out loud the number of fruits separately for each fruit category (orange, apple, and pear). They were instructed not to move towards the fruit before starting to say the counting value. The participants started each block counting from zero. The appearance of the fruit categories was randomized with the only condition that each block should contain one fruit of each three categories, except for the first and last blocks, which only contained pears.

#### Virtual environment

The VE displayed in the 2D screen and IVR conditions was composed of a virtual representation of the room, including the walls, the ceiling with a lamp, the ground, the door, the curtain, and the table (Fig. 1e). Respecting the physical light location, shadows were cast from the avatar, the virtual HTC Vive controller held in the right hand, and the virtual HTC Vive trackers on the arm. The blue sphere (touching point), green sphere (workspace center), and the fruits did not cast shadows as it was not possible to render those shadows with the Meta 2 AR display and we wanted to have a fair comparison between technologies. In the AR condition, only the spheres and fruits were rendered and lit by the same light sources used in the other VE. A black and unlit controller was also rendered on top of the real one for occlusion purposes; the default occlusion algorithm of the Meta 2 worked rather well for detecting participants’ hands, but not the controller, probably due to its material.

The avatar’s arm was animated with inverse kinematics (IK) using the tracked position and orientation of the controller held in the participant’s hand. Only in the IVR condition, the avatar’s neck and spine were animated with IK based on the orientation of the HMD. The whole avatar’s position was also adapted in the 3D space to match the tracked position of the HMD.

#### Protocol

**Fig. 2:**
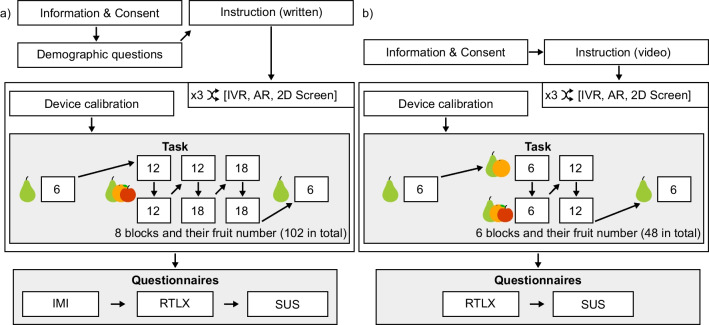
Experimental protocols. **a** Experiment 1 with elderly participants; **b** Experiment 2 with brain-injured patients

A visual representation of the experimental protocol is shown in Fig. 2a. Each participant performed the same motor and cognitive tasks under the three different visualization technology conditions (IVR, AR, and 2D screen). The order of the conditions was balanced between all participants. In each visualization condition, we aimed at displaying a similar environment (i.e., experiment room), which required a similar interaction (i.e., moving in 3D with a tracked controller).

Before the experiment started, participants sat on a comfortable chair, answered the demographic questionnaire (see Table 1), and received the tasks instructions orally. Before each condition, a calibration of the corresponding visualization technology was performed (see section Experimental setup). After all conditions were performed, participants filled in the following questionnaires: the “Raw Task Load Index” (RTLX [32]) to evaluate the subjectively reported cognitive load (with subscales “Mental Demand”, “Physical Demand”, “Temporal Demand”, “Performance”, “Effort”, and “Frustration”); several items from the “Intrinsic Motivation Inventory” (IMI [33]) to measure the motivation (with subscales “Interest/Enjoyment”, “Perceived Competence”, “Effort/Importance”, and “Pressure/Tension”); and the “System Usability Scale” (SUS [34]) to evaluate the system usability.

The questionnaires were answered on a computer using REDCap electronic data capture tool [35] hosted at the University of Bern, Switzerland. For each question, three lines of answers were possible—one per visualization condition, respecting the order of appearance of each display. The SUS and IMI were answered using a Likert scale between 1 and 7 points; 1 indicating “Not at all”, 4 indicating “Somewhat true”, and 7 indicating “Very true”. The RTLX used a markerless slider without numerical values with 100 encoded intervals. All questions were translated into German.

#### Data processing

##### Questionnaire﻿﻿s

 A single score was computed for the SUS questionnaire by averaging all questions and rescaling from the original 1–7 Likert scale to 0–100. For the IMI, a single value per subscale was computed by averaging all the questions within each subscale, 5–7 questions per subscale. For the RTLX, we used the selected values on the markerless slider (from 0 to 100) for the six questions, one question per subscale.

##### Cognitive Task

For the counting task, a score was computed for each block as the percentage of correct counted fruits over the total of presented fruits in that block. If a mistake was made while counting a fruit, participants could continue counting from this erroneous value—i.e., if the expected number was three, but the participant said four, both four and five would be considered as a correct next counting value. The percentages calculated for each of the eight blocks within a condition were averaged into a single value for each participant.

##### Movement Quality

The position and orientation of the HTC controller were recorded at an approximate frequency of 500 Hz. The data was cut into individual fruit-reaching movements, starting from the instant that the green sphere disappeared and the fruit appeared and ending when the fruit disappeared, i.e., when the blue sphere collided with the fruit collider.

Four movement quality metrics were computed from the blue sphere location to evaluate the movement quality: (1) The **normalized movement duration** (s/m), defined as the duration of the reaching movement divided by the minimum distance between the last and first position within the movement; (2) The **trajectory straightness ratio** (n.u.), defined as the length of the path followed divided by the minimum distance between the last and first position within the movement; (3) The **peak velocity** (m/s) defined as the highest velocity value during the movement; and (4) The **number of velocity peaks**—reflecting the movement smoothness [36].

We also calculated the **movement onset**, defined as the time lapsed between the disappearance of the green sphere and the instant the speed of the blue sphere reached the threshold of 0.2 m/s.

The data were processed in *Python* 3.7.9, with the packages *numpy* 1.20.2, *pandas* 1.1.3, *quaternion* 2021.4.5.14.42.35, and *scipy* 1.5.2.

#### Statistical analyses

As we expected that the quality of the reaching movements requiring depth would decline in the 2D screen condition, we classified the reaching movements within three categories, based on the fruit location (**depth usage**): (1) *no depth*, i.e., only movements along the horizontal and/or vertical axes were needed; (2) *depth only*, i.e., no movements along the horizontal nor the vertical axes were needed; and (3) *combined depth*, i.e., the fruit location required movements in the depth axis along with the horizontal and/or vertical axes.

To investigate the impact of the *visualization technology* (IVR, AR, 2D screen) and the *depth usage* (no depth, depth only, combined depth) and their interaction on the four movement quality metrics and movement onset, we performed a two-way 3 x 3 RM-ANOVA. None of the movement quality metrics followed a normal distribution, based on Kolmogorov-Smirnov tests. However, in the absence of non-parametric alternatives, we decided to use the RM-ANOVA knowing that our analyses were, therefore, more conservative than non-parametric tests.

To investigate the impact of the visualization technology on the motivation (IMI) and reported cognitive load (RTLX), we computed an averaged value per subscale, following each specific questionnaire convention. For each questionnaire, we performed a one-way repeated-measures multivariate analysis of variance (RM-MANOVA) considering the visualization technology as an independent variable and the subscales of the questionnaires as dependent variables. For the system usability (SUS), as it has no defined subscales, we performed a one-way repeated-measures analysis of variance (RM-ANOVA) on the average of all questions. Only the IMI had one extreme outlier ($outlier < Q_1-3\cdot IQR$ or $outlier > Q_3+3 \cdot IQR$; $Q_1$: first quartile, $Q_3$: third quartile, and $IQR = Q_3-Q_1$). Removing this participant and performing the RM-MANOVA again led to similar results, therefore, the reported values include this participant.

To analyze the impact of the visualization technology on the counting accuracy—the objective measure of cognitive load—, we ran a Friedman test as the Kolmogorov-Smirnov test indicated a normality violation for the counting accuracy in IVR.

When a significant main effect of a factor or an interaction was found, post-hoc pairwise *t*-tests were performed and the *p*-values adjusted for multiple hypothesis testing using Bonferroni correction. We applied the Greenhouse-Geisser sphericity correction for factors violating the sphericity assumptions in the RM-MANOVA and RM-ANOVA tests. The reported effect sizes for the RM-MANOVA and RM-ANOVA tests are the partial $\eta ^2$. We reported the Cohen’s D for the post-hoc tests, and for the Friedman test, the Kendall’s coefficient of concordance (W).

Three participants (two females, one male) were excluded from the statistical analyses. For two participants, we encountered technical problems with the AR HMD device. The third exclusion was due to the inability of the participant to stay in contact with the green sphere between each fruit reach in the 2D screen condition. For each metric, we excluded extreme outliers of each participant ($< Q_1-3\cdot IQR$ or $> Q_3+3\cdot IQR$; $Q_1$: first quartile, $Q_3$: third quartile, and $IQR = Q_3-Q_1$). Over the total of 5886 reaching movements performed by the 17 participants in all visualization conditions, the extreme outliers removal led to 660 movements removed from the movement onset computation (IVR: 164, AR: 230, 2D screen: 266; 280 of them did not reach the minimum velocity threshold), 140 from the normalized movement duration (IVR: 55, AR: 56, 2D screen: 29), 135 from the trajectory straightness ratio (IVR: 35, AR: 72, 2D screen: 28), 51 from the peak velocity (IVR: 9, AR: 14, 2D screen: 28), and 174 from the number of velocity peaks (IVR: 43, AR: 83, 2D screen: 48).

The RM-MANOVAs and their univariate follow-up tests were performed in *SPSS* version 27. The RM-ANOVAs and the post-hoc tests were performed using *Python* 3.7.9, with the packages *numpy* 1.20.2, *pandas* 1.1.3, *r-afex* 0.23_0, *r-effsize* 0.7.6, *rpy2* 2.9.4, *scipy* 1.5.2, and *statsmodels* 0.12.2. The significance level was set to $\alpha = 0.05$ for all statistical tests.

### Experiment 2—brain-injured patients

#### Participants

Five participants with moderate motor impairment due to a neurologic incident, in the subacute phase (< two months after the incident) and aged 36 to 69 (49.88 ± 12.55) participated in the second experiment. Patients were screened by a clinician for the following inclusion criteria: (1) motor impairment due to brain-injury, (2) able to move the affected arm with weight support (i.e., enrolled in the physical therapy sessions using Armeo $\circledR$ Spring (Hocoma, Switzerland) at the hospital), and (3) no severe visual or auditory impairments (strabismus, macular degeneration, retinopathy). The use of glasses or contact lenses was allowed during the experiment. The clinical data of the participants can be found in Table 2. They were recruited by therapists from the rehabilitation unit of the University Hospital Bern, Switzerland. Participants provided written informed consent to participate in the study. The study was approved by the local Ethics Committee (ref.: 2017-02,195) and conducted in compliance with the Declaration of Helsinki. Participants did not receive any compensation for their participation in the study.

**Table 2:** Patients’ characteristics

#	Gender	Age	Lesion type	Lesion location	Time since onset (days)	Sensori- motor hemi- paresis side	Aphasia	Chedoke McMaster Hand assessment score$^{1}$	Neuro- psychological deficits
1	Female	36	Hemorrhagic stroke	Paracentral left	34	Right	No	5	Reduced digital span and verbal fluency
2	Male	38	Epilepsy surgery	Resection of the right operculum	28	Left	No	7	Attention, visuo- constructive, and executive deficits
3	Male	69	Ischemic stroke	Right frontoparietal	48	Left	No	5	Left hemineglect
4	Male	58	Hemorrhagic stroke	Left basal ganglia	35	Right	Yes	4	Attention and executive deficits. Reduced resilience
5	Male	48	Ischemic stroke	Arteria cerebri media right	33	Left	No	4	Left hemineglect, dysexecutive symptoms, perseveration, visuo- constructive disturbances

$^{1}$ Test performed within 1 week before or after the experiment

#### Experimental setup and virtual environments

Since patients suffered moderate motor impairments, we adapted the fruit-reaching motor task so the VR task could be interfaced with the Armeo $\circledR$ Spring (Hocoma, Switzerland) to provide arm weight support during the experiment. The weight support system and VR game interfaced using User Datagram Protocol (UDP) communication, which was provided by Hocoma, Switzerland. The task was implemented to be feasible both with the left or right arm, so patients could always perform it with their paretic arms.

We noticed that the hand/device end-effector position obtained by the UDP communication from the ArmeoSpring—calculated from the device position sensors—was not precise enough, i.e., there was a visible offset between the real hand position and the one rendered in AR using the device forward kinematics calculations. To reduce this visual mismatch, we included three *HTC Vive trackers (2018)* to track several links of the mechanical structure. A first tracker was placed on the height-adjustable part of the ArmeoSpring, which is used to adjust the height of the device based on the patient’s height and is fixed during the training (Fig. 1b; fixed reference tracker). Its location was, therefore, considered as a fixed reference frame to our system, from which we were able to compute the patient’s seated position (assuming a fixed position shift from the tracker to the closest point between the two shoulders, visible in Fig. 1d). The second tracker was placed on the ArmeoSpring upper arm link at the shoulder level to track the location of the patient’s shoulder. This was needed as the device allows shoulder movements on the sagittal plane, but does not incorporate sensors to measure those. Finally, the third tracker was mounted with an in-house 3D-printed fixation element on the ArmeoSpring hand module to track the patient’s hand location. The avatar’s arm was then animated using the *Unity* plugin *FinalIK* v1.9. We used the tracker on the hand module to compute the hand position and the tracker next to the shoulder to compute the root position of the avatar's arm. Finally, the elbow position was computed using the FinalIK algorithm with the UDP ArmeoSpring sensor data as a hint for the elbow location.

To facilitate the recruitment of brain-injured patients, the experiment was performed in a room at the University Hospital Bern (Inselspital), different than the one used in the first experiment, which was performed at the Swiss Institute for Translational and Entrepreneurial Medicine (SITEM-Insel). The room had artificial and controllable lighting. To remove background details that could interfere with the visibility of the fruits in the AR condition, a black board was placed in front of the participants and behind the task workspace. The virtual reproduction of the room included only four walls, the ceiling, the ground, and the black background board (Fig. 1f). The virtual light source within the VE used the same location as the real one and was also employed to compute the lighting on the virtual elements in the AR modality. No calibration was needed for the 2D screen modality.

The avatar rendered in the IVR and 2D screen conditions held a vertical black cylinder (corresponding to the real ArmeoSpring hand module; Fig. 1f). In all conditions (also in AR), a white virtual horizontal cylinder was added to the hand module. A virtual blue sphere of 4 cm in diameter was attached at the end of this white cylinder. These virtual elements were included to preserve the distance from the patient’s hand location and the touching point in Experiment 1 due to the length of the HTC Vive controller.

#### Protocol & motor and cognitive tasks

The protocol of Experiment 2 is depicted in Fig. 2b. The protocol was similar to the one described in Experiment 1, with only minor differences to reduce the duration and task difficulty. First, there was no demographic questionnaire at the start of the experiment, as the most relevant information (Table 2) was provided by the therapists with the patient’s consent. Second, the oral instructions were supported with a video to show the task to be performed and the different visualization conditions. Third, the AR calibration step was not performed because the therapists and medical doctors considered it too demanding for the neurologic patients. Fourth, to shorten the whole experiment, we did not include the motivation questionnaire, as it was the longest questionnaire. The scale of the usability questionnaire was changed from a 7-point to a 5-point Likert scale. Finally, to facilitate the understanding of the cognitive load and usability questionnaires (RTLX & SUS), those were provided in paper form—instead of using REDCap with a computer. We included photos of the different displays to help identifying the different conditions and, when needed, the assistance of the experimenter was provided. The order of the questionnaires was balanced between the patients, and the order of the items within each questionnaire was randomized for each patient.

The motor and cognitive tasks were very similar to the ones performed in Experiment 1, with three exceptions. First, we adjusted the difficulty of the tasks by defining only six blocks of, respectively, 6, 6, 6, 12, 12, and 6 fruits, i.e., a total of 48 fruits per condition. The first and last blocks only contained pears, the second and fourth blocks only contained pears and oranges, and the third and fifth block contained the three fruit categories. Second, the diameter of the green sphere was increased from 4 cm to 5 cm to be more tolerant to errors. Third, the workspace had the same size but its center was centered on the left-right axis, and located 31 cm down, and 40 cm away from the participants’ eyes to fit a space easily reachable by the patients.

#### Data processing

A single score, rescaled from the original 1–7 Likert interval to 0–100, was computed for the SUS by averaging all the questions. For the RTLX, we used an analogical scale of 122–125.5 mm (variations due to printer inconsistency) with 21 interval marks. The value of each RTLX answer was calculated as the distance from the left border to the centers of the participants’ added responses (crosses) over the analogical scale (rounded to the closest 0.5 mm), divided by total physical scale size— i.e., 122–125.5 mm —and multiplied by 100. We used the same procedure as in Experiment 1 to compute the cognitive task score.

We computed the same four movement quality metrics and the movement onset for each (fruit) reaching movement as in Experiment 1, using the recorded position and rotation of the hand module tracker to compute the blue sphere location. We categorized the movements using the same depth usage classification as in Experiment 1.

We followed the same procedure to find and remove movement outliers for each patient. Over the total of 720 individual reaching movements, the outliers removal led to 164 movements removed from the movement onset computation (IVR: 46, AR: 48, 2D screen: 70; 144 of them did not reach the minimum speed threshold), 15 from the normalized movement duration (IVR: 8, AR: 4, 2D screen: 3), three from the trajectory straightness ratio (IVR: 1, AR: 1, 2D screen: 1), six from the peak velocity (IVR: 2, AR: 1, 2D screen: 3), and 14 from the velocity peaks number (IVR: 4, AR: 3, 2D screen: 7). Movement outliers were distributed across patients and conditions and did not predominantly affect a single patient.

As the number of patients was relatively low, we did not have enough statistical power to perform statistical analyses. Therefore, we only report the mean and standard deviation for each metric.

## Results

### Experiment 1: healthy elderly participants

**Fig. 3:**
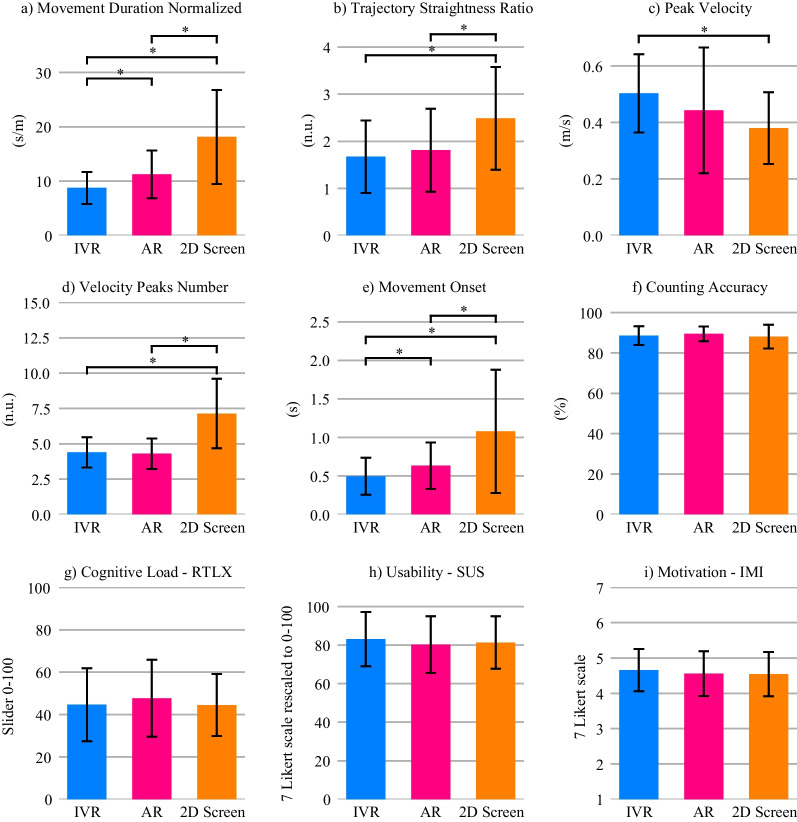
Effects of the visualization technologies in the healthy elderly participants (Experiment 1) on: **a**–**d** Movement quality, **e** Movement onset, **f** Cognitive load with parallel counting task, **g** Self-reported cognitive load, **h** Usability, and **h** Motivation. Error bars: ± 1 SD. * $p < 0.05$

**Fig. 4:**
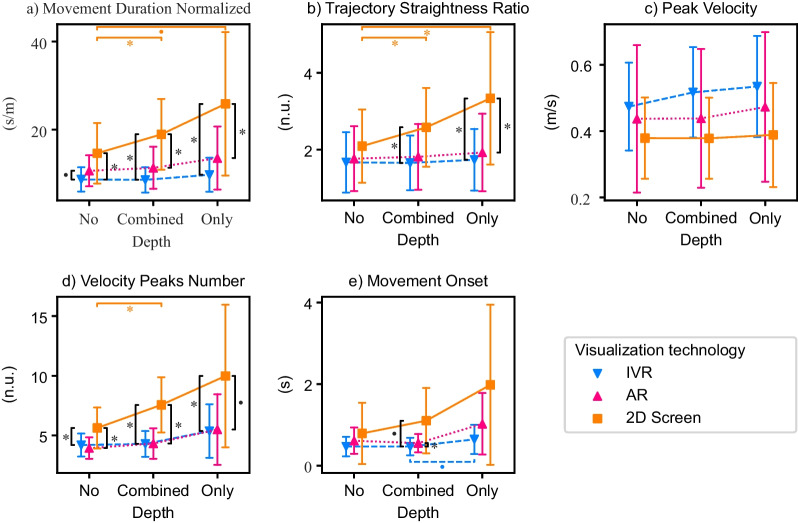
Interaction effects between the visualization technology and depth usage in healthy elderly participants (Experiment 1) on: **a**–**d** Movement quality and **e** Movement onset. Visualization technologies: IVR, AR, 2D Screen. Depth usage: No (movements along the horizontal and/or vertical axes), Only (movements only along the depth axes), Combined (movements in the depth axis along with the horizontal and/or vertical axes). Error bars: ± 1 SD. * $p < 0.05$, $\bullet$ $p < 0.1$

The results of the RM-MANOVAs, RM-ANOVAs, and Friedman tests for the self-reported questionnaire values—i.e., motivation (IMI), cognitive load (RTLX), and usability (SUS)—, the movement quality metrics—i.e., normalized movement duration, trajectory straightness ratio, peak velocity, and velocity peaks number—, the movement onset, and the counting accuracy can be found in Table 3. The results of the follow-up analyses are summarized in Table 4. The impact of the visualization technology on the different metrics is graphically represented in Fig. 3 and the interaction effect between the visualization technology and the depth usage on movement quality and movement onset in Fig. 4.

**Table 3:** Experiment 1 results from the RM-MANOVAs on the effect of the visualization technology (*Vis. Tech.*) on the questionnaire data, the results from the RM-ANOVAs on the effect of the visualization technology, depth usage (*Depth*) and its interaction (*Vis. Tech.:Depth*) on the movement quality and movement onset, and the Friedman test on the counting accuracy

Effect	df	F	Effect size	Sig.
RM-MANOVA—Motivation (IMI)				
Vis. Tech.	4	.79	.41	.627
RM-MANOVA—Cognitive Load (RTLX)				
Vis. Tech.	6	.66	.61	.745
RM-ANOVA—Usability (SUS)				
Vis. Tech.	2	2.42	.95	.105
RM-ANOVA—Normalized duration				
Vis. Tech.	2	26.4	.7	< .001 *
Depth	2	11.28	.83	.003 *
Vis. Tech.:Depth	4	9.0	.84	.003 *
RM-ANOVA—Trajectory straightness ratio				
Vis. Tech.	2	12.43	.74	< .001 *
Depth	2	14.98	.94	.001 *
Vis. Tech.:Depth	4	18.54	.94	< .001 *
RM-ANOVA—Peak velocity				
Vis. Tech.	2	7.0	.78	.012 *
Depth	2	6.17	.98	.016 *
Vis. Tech.:Depth	4	2.39	.97	.12
RM-ANOVA—Velocity peaks number				
Vis. Tech.	2	24.97	.68	< .001 *
Depth	2	9.88	.65	.005 *
Vis. Tech.:Depth	4	4.98	.73	.012 *
RM-ANOVA—Movement onset				
Vis. Tech.	2	11.21	.61	.003 *
Depth	2	8.29	.64	.01 *
Vis. Tech.:Depth	4	3.49	.61	.07 $\bullet$

Effect	df	$\varvec{\chi }^2$(2)	Effect size	Sig.
Friedman test—Counting accuracy				
Vis. Tech.	2	.27	.008	.874

For the RM-MANOVAs, the reported *F values* are the Wilks' $\lambda$

* $$p < 0.05$$, $$\bullet$$ $$p < 0.1$$

**Table 4:** Experiment 1 results from the post-hoc tests

Group1	Group2	T	Effect size	Sig.
Normalized duration				
IVR	AR	− 3.47	0.61	0.009 *
IVR	2D Screen	− 5.87	0.94	< 0.001 *
AR	2D Screen	− 4.83	0.77	0.001 *
Combined	No	3.31	− 0.33	0.013 *
Combined	Only	− 3.04	0.31	0.023 *
No	Only	− 3.34	0.58	0.012 *
AR & Combined	2D Screen & Combined	− 5.39	0.97	0.002 *
AR & Only	2D Screen & Only	− 4.24	0.69	0.022 *
2D Screen & Combined	2D Screen & No	4.97	− 0.53	0.005 *
2D Screen & No	2D Screen & Only	− 3.62	0.67	0.082 $\bullet$
IVR & Combined	2D Screen & Combined	− 6.63	1.16	< 0.001 *
IVR & No	AR & No	− 3.57	0.57	0.093 $\bullet$
IVR & No	2D Screen & No	− 4.96	0.68	0.005 *
IVR & Only	2D Screen & Only	− 4.63	0.89	0.01 *
Straightness ratio				
AR	2D Screen	− 3.15	0.67	0.019 *
IVR	2D Screen	− 4.91	0.79	0.001 *
Combined	No	4.03	− 0.22	0.003 *
Combined	Only	− 2.86	0.24	0.034 *
No	Only	− 4.11	0.41	0.003 *
AR & Only	2D Screen & Only	− 4.86	0.82	0.006 *
2D Screen & Combined	2D Screen & No	6.04	− 0.47	0.001 *
2D Screen & No	2D Screen & Only	− 5.05	0.59	0.004 *
IVR & Combined	2D Screen & Combined	− 5.75	0.92	0.001 *
IVR & Only	2D Screen & Only	− 5.12	0.9	0.004 *
Peak velocity				
IVR	2D Screen	5.23	− 0.92	< 0.001*
No	Only	− 2.82	0.22	0.037 *
Velocity peaks number				
AR	2D Screen	− 6.52	1.12	< .001 *
IVR	2D Screen	− 5.52	1.25	< .001 *
Combined	No	3.62	− 0.63	0.007 *
Combined	Only	− 2.73	0.35	0.045 *
No	Only	− 3.19	0.79	0.017 *
AR & Combined	2D Screen & Combined	− 7.72	1.44	< 0.001 *
AR & No	2D Screen & No	− 4.59	1.09	.011 *
AR & Only	2D Screen & Only	− 3.85	0.79	0.051 $\bullet$
2D Screen & Combined	2D Screen & No	4.44	− 0.88	0.015 *
IVR & Combined	2D Screen & Combined	− 5.86	1.67	0.001 *
IVR & No	2D Screen & No	− 4.49	0.87	0.013 *
IVR & Only	2D Screen & Only	− 3.87	0.77	0.049 *
Movement onset				
IVR	AR	− 3.73	0.46	0.005 *
IVR	2D Screen	− 3.71	0.67	0.006 *
AR	2D Screen	− 3.03	0.52	0.024 *
Combined	Only	− 3.4	0.58	0.011 *
No	Only	− 3.22	0.8	0.016 *
IVR & Combined	IVR & Only	− 3.66	0.41	0.076 $\bullet$
IVR & Combined	AR & Combined	− 4.01	0.37	0.036 *
IVR & Combined	2D Screen & Combined	− 3.75	0.73	0.063 $\bullet$

Visualization technologies: IVR, AR, 2D Screen. Depth usage: No (movements along the horizontal and/or vertical axes), Only (movements only along the depth axes), Combined (movements in the depth axis along with the horizontal and/or vertical axes)

$$* p< 0.05, \bullet p < 0.1$$

#### Movement quality

With the IVR technology, elderly healthy participants performed movements of shorter duration compared to the other two visualization technologies (Fig. 3a). Visualizing the movements in IVR also resulted in straighter, faster, and smoother movements (i.e., less number of velocity peaks) than with the 2D screen (Fig. 3b–d). With AR, the reaching movements were of shorter duration, straighter, and smoother compared to the 2D screen (Fig. 3a,b,d).

Movements that required moving along the depth axis (either only along the depth axis or in combination with another dimension) were in general of longer duration, less straight, and less smooth than movements that did not incorporate depth at all (Table 4). Furthermore, movements that only required moving along the depth axis were also of longer duration, less straight, and less smooth than movements combining depth with another dimension.

We also found significant interaction effects between the visualization technology and the depth usage in all movement quality metrics, except in the peak velocity (Table 3). Post-hoc tests revealed that, for the 2D screen, the reaching movements were of shorter duration, straighter, and smoother when there was no depth component compared to the combination of depth with another dimension (Table 4, Fig. 4). The movements were also straighter when no depth was used compared to movements along only the depth axes (Fig. 4b). When comparing the same depth usage between different technologies, we found that when no depth was used, IVR led to shorter duration and smoother movements than the 2D screen and a trend also indicated that they were of shorter duration than with AR (Fig. 4a, d). When only depth was used, both HMDs led to shorter duration and straighter movements than the 2D screen (Fig. 4a, b). The 2D screen also led to less smooth movements than IVR and a trend indicated less smooth movements than AR (Fig. 4d).

#### Movement onset

With the IVR HMD, participants performed reaching movements towards the fruits that started earlier compared to the two other technologies—i.e., smaller movement onset (Fig. 3e). With AR, the movements also started earlier compared to the 2D screen.

The onsets of reaching movements requiring only the depth dimension were longer than those movements that combined depth with another dimension, or not using depth at all (Table 4).

We found that the interaction effect between the visualization technology and the depth usage in the movement onset did not reach significance ($p = 0.07$; Table 3). Nevertheless, we decided to run post-hoc tests to have a closer look at potential differences (Table 4). Post-hoc tests revealed that when comparing different depth usages within the same visualization technology, there was a trend within the IVR technology, indicating that movements using only depth started later than the ones combining depth with another dimension. Within the same depth usage, we found that, for locations combining depth with another dimension, IVR led to movements starting earlier than AR and a trend indicated that they started earlier than the 2D screen (Fig. 4e).

#### Counting task accuracy and questionnaires

The overall reported usability was high (> 80 over a maximum of 100) with every visualization technology and did not differ significantly across them (Fig. 3h). The reported motivation was also relatively high (> 4.5 over a maximum of 7)—considering the fact that the task was not designed to enhance motivation—and did not differ significantly across visualization technologies (Fig. 3i). The self-reported cognitive load—measured with the RTLX questionnaire—did not differ significantly across visualization technologies (Fig. 3g). We did not find significant differences either in the counting accuracy in the parallel cognitive task, which remained high across technologies (> 80 over a maximum of 100) (Fig. 3f).

### Experiment 2: brain-injured patients

**Fig. 5:**
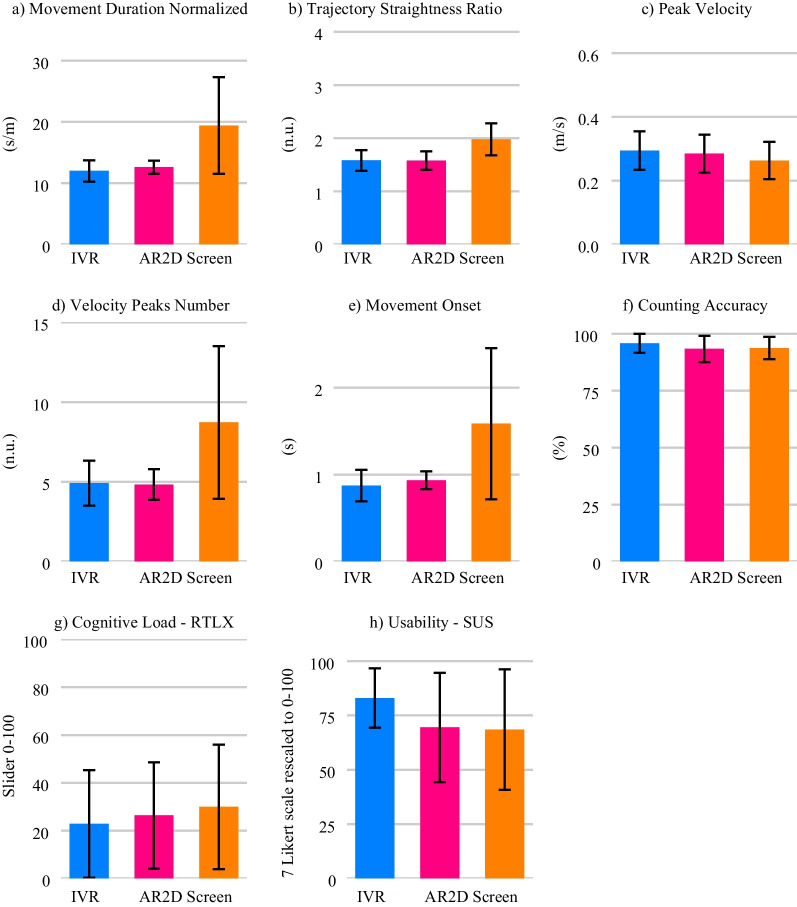
Effect of the visualization technologies in the five brain-injured patients (Experiment 2) on: **a**–**d** Movement quality, **e** Movement onset, **f** Cognitive load with parallel counting task, **g** Self-reported cognitive load, and **h** Usability. Error bars: ± 1 SD

**Fig. 6:**
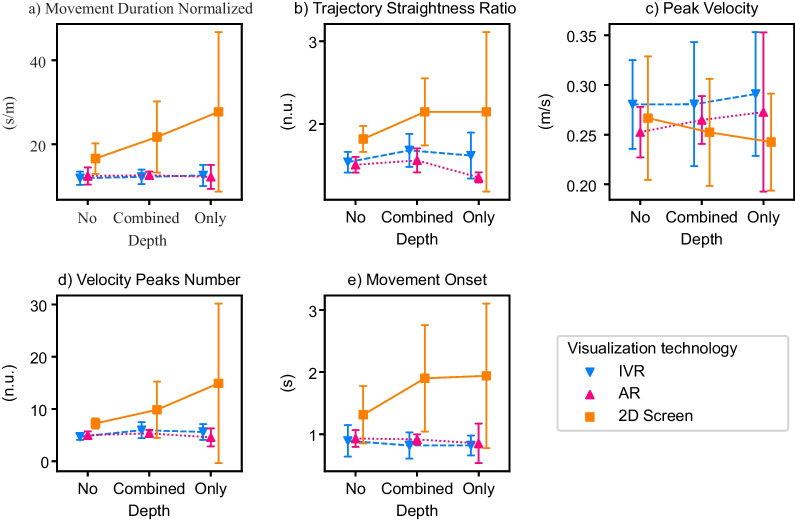
Interaction effects between the visualization technology and depth usage in brain-injured patients (Experiment 2) on: **a**–**d** Movement quality and **e** Movement onset. Error bars: ± 1 SD

A summary of the descriptive statistics of the movement quality metrics, movement onset, counting task accuracy, self-reported cognitive load (RTLX), and usability (SUS) under the three different visualization technologies can be found in Table 5 and a graphical representation is available in Fig. 5. A summary of the descriptive statistics detailed by the visualization technology and the depth usage on the movement quality and movement onset can be found in Table 6 and a graphical representation is available in Fig. 6.

**Table 5:** Descriptive statistics across visualization technologies in Experiment 2

Variable	IVR	AR	2D Screen
Movement duration normalized (s/m)	11.99 (± 1.73)	12.6 (± 1.1)	19.39 (± 7.89)
Trajectory straightness ratio (n.u.)	1.58 (± 0.19)	1.58 (± 0.17)	1.98 (± 0.3)
Peak velocity (m/s)	0.29 (± 0.06)	0.28 (± 0.06)	0.26 (± 0.06)
Velocity peaks number (n.u.)	4.92 (± 1.41)	4.83 (± 0.97)	8.72 (± 4.79)
Movement onset (s)	0.87 (± 0.18)	0.93 (± 0.1)	1.58 (± 0.87)
Counting accuracy (%)	95.83 (± 4.17)	93.33 (± 5.78)	93.75 (± 4.89)
Cognitive load—RTLX$^{1}$	22.81 (± 22.5)	26.35 (± 22.21)	29.95 (± 26.02)
Usability–SUS$^{2}$	83.0 (± 13.62)	69.5 (± 25.15)	68.5 (± 27.7)

Mean values and standard deviations of the movement quality, movement onset, cognitive load with a parallel task, self-reported cognitive load, and usability across visualization technologies for the brain-injured patients

$$^{1}$$ Slider 1–100

$^{2}$ 7 Likert scale rescaled to 0-100

**Table 6:** Descriptive statistics across visualization technologies and depth usage in Experiment 2

Variable	Visualization technology	No depth	Combined depth	Only depth
Movement duration normalized (s/m)	IVR	11.88 (± 1.59)	11.89 (± 1.9)	12.59 (± 2.52)
	AR	12.95 (± 2.29)	12.18 (± 1.36)	11.88 (± 2.98)
	2D Screen	15.89 (± 3.99)	20.94 (± 8.67)	25.4 (±1 9.67)
Trajectory straightness ratio (n.u.)	IVR	1.5 (± 0.15)	1.63 (± 0.23)	1.52 (± 0.35)
	AR	1.63 (± 0.29)	1.57 (± 0.15)	1.35 (± 0.06)
	2D Screen	1.78 (± 0.18)	2.1 (± 0.42)	2.1 (± 0.97)
Peak Velocity (m/s)	IVR	0.29 (± 0.05)	0.3 (± 0.07)	0.28 (± 0.07)
	AR	0.28 (± 0.07)	0.29 (± 0.05)	0.29 (± 0.09)
	2D Screen	0.28 (± 0.07)	0.25 (± 0.05)	0.24 (± 0.05)
Velocity peaks number (n.u.)	IVR	4.33 (± 1.0)	5.37 (± 1.97)	4.97 (± 2.05)
	AR	4.8 (± 0.83)	4.92 (± 1.12)	4.45 (± 1.74)
	2D Screen	6.7 (± 1.52)	9.06 (± 5.66)	12.72 (± 16.03)
Movement onset (s)	IVR	0.9 (± 0.25)	0.83 (± 0.21)	0.87 (± 0.19)
	AR	0.94 (± 0.14)	0.93 (± 0.09)	0.85 (± 0.32)
	2D Screen	1.24 (± 0.49)	1.72 (± 0.95)	1.88 (± 1.17)

Mean values and standard deviations of the movement quality and movement onset across visualization technologies and depth usage for the brain-injured patients

#### Movement quality

We observed that both IVR and AR HMDs seemed to lead to shorter duration, straighter, and smoother movements compared to the 2D screen (Fig. 5). Another interesting observation was that the standard deviation of those metrics was much smaller with the HMDs than with the 2D screen. The reaching movements seemed also to reach higher velocity peaks with both HMDs compared to the 2D screen, but the differences in this specific metric seemed smaller than in the other movement quality metrics.

Regarding depth usage (Fig. 6), similar to what we observed in the elderly participants, only the 2D screen seemed to elicit longer duration, less straight, and less smooth movements when the reaching towards fruits did require moving in the depth axis compared to movements requiring no depth. None of the HMDs seemed to be impacted by depth usage. As with the healthy elderly participants, there seemed to be no interaction effect on the peak velocity, although we observed a slight decrease in the peak velocity in the 2D screen when moving in the depth axis was required, which was not observed in the HMDs.

#### Movement onset

We observed that both HMDs seemed to lead to smaller movement onsets with smaller standard deviations compared to the 2D screen (Fig. 5e). No differences in the movement onset were, at first glance, observed between the AR and IVR visualizations. We also observed that the movement onset was larger when the reaching movements required moving in the depth axis (compared to no depth) only with the 2D screen (Fig. 6e).

#### Counting task accuracy and questionnaires

For the counting task accuracy, no apparent differences were observed between visualization technologies (Fig. 5f). However, the reported cognitive load in the RTLX questionnaire seemed to be higher with the 2D screen than with the HMDs (Fig. 5g). The self-reported cognitive load also seemed to be smaller with the IVR compared to the AR. Finally, regarding the usability, IVR was reported as the most usable, with a remarkable high value of 83, even higher than the average value reported by the elderly participants (Table 5, Fig. 5h). The AR and the 2D screen visualizations showed a lower usability with a high between-subject standard deviation.

## Discussion

In this study, we investigated whether IVR and AR HMDs could improve movement quality, reduce cognitive load, and increase motivation and usability compared to a 2D screen using parallel motor and cognitive tasks—i.e., reaching towards and counting virtual fruits, respectively—and questionnaires. We also analyzed whether the visualization technology impacted differently the movement quality and movement onset depending on the depth usage requirements of the reaching movements. We performed a first experiment with 20 elderly participants using a VR controller and a second pilot experiment with five brain-injured patients. For this second experiment, we adapted the experimental setup to be used in combination with a rehabilitation device (Armeo$\circledR$ Spring, Hocoma, Switzerland).

### HMDs improve the movement quality

As hypothesized, the movement quality improved with HMDs compared to the 2D screen. The improvement in movement quality could be observed in all movement quality metrics, i.e., reaching with the HMDs resulted in shorter duration, faster, straighter, and smoother movements. The differences between HMDs and the 2D screen reached significance in the elderly participants, while similar differences were observed in the smaller group of brain-injured patients. Contrary to our previous experiment with young healthy participants [23], AR showed a significant increase in the movement quality over the 2D screen in the elderly participants. The first observations in the patient population also seem to go in the same direction. Nevertheless, IVR still appears to surpass AR, as the movements were of shorter duration with IVR than with AR, at least in the elderly participants.

We expected that, only in the 2D screen condition, reaching movements would worsen when they involve the depth dimension—i.e., when the movements were only on the depth dimension or when they involved both horizontal/vertical movements together with the depth dimension. We expected that the depth dimension would be harder to visualize than the horizontal and vertical dimensions, which are directly mapped to the 2D screen plane. Our results confirm that, indeed, only in the 2D screen condition did the movement quality degrade when the depth dimension was required in the reaching movement (in terms of movement duration, straightness, and smoothness). The differences were more obvious between the movements with no depth vs. movements that required only moving in the depth axes than movements combining depth and the vertical/horizontal directions.

These analyses over the depth usage complete our previous “dimensionality” analyses with young healthy participants [23]. In our previous analyses, we compared the movement quality between visualization technologies based on the number of dimensions of the reaching movement, i.e., 1D, 2D, and 3D, instead of the use of depth dimensions. For example, the “1D” movements contained movements not using the depth dimension but also some using it only. This might explain why we found more interaction effects between the visualization technologies and depth usage in the current study compared to those in the previous experiment. Our results differ from those of the study of Gerig et al. [25] where the quality of reaching movements was compared between IVR and 2D screens. In their study with healthy participants, the authors reported shorter, straighter, and smoother movements in IVR than with a 2D screen with limited depth cues only when the 2D screen did not show a known-size object (i.e., the HTC controller) as an additional depth cue. In our experiment, the differences between modalities where significant, even if the controller was visually represented in the VE of both IVR and 2D screen modalities. However, we note that other depth cues (e.g., the shadows of the targets) were not present in our design as the selected optical see-through AR HMD could not render them, and we aimed to have a fair comparison between the technologies. Furthermore, in [25], the study population was also younger (18–40 years old) than our elderly population (64–89 years old) and, possibly, more cognitively fit.

Our results are encouraging for the adoption of HMDs into VR-based neurorehabilitation interventions. As identified by Palacios-Navarro and Hogan in their recent review [26], there is a lack of studies investigating how improved depth perception in immersive VR using HMDs could improve VR-based interventions in upper limb rehabilitation. In the real world, many movements involve moving toward the depth direction, and therefore, it is important to train in an environment that alters as little as possible the perception and execution of such movements [6, 19, 37]. Using HMDs allow the provision of congruent sensory information between vision and proprioception, which—as observed in our experiment—enhances movement quality, but could also promote neuroplasticity by allowing meaningful movement training that promotes multi-sensory input to the central nervous system [11]. Furthermore, this congruent sensory information might enhance skill transfer into ADL [6], as differences in depth perception might be associated with a low transfer of learned skills observed when training in non-immersive VR [38].

In the field of robotic rehabilitation, where the interaction with the rehabilitation system differs from real life, it is an open question whether patients relearn to use their arm or adapt to the training system (e.g., robotic device or visualization technology) [39]. Providing more naturalistic depth cues and reducing the visuospatial transformation with HMDs is a promising way to avoid the observed two-step learning in robotic VR-based interventions, where patients first go through a phase of learning how to use the system before focusing on their rehabilitation [20]. Therefore, the use of HMDs might allow patients to immediately train the targeted functional movements, gaining crucial rehabilitation time.

Finally, as movement quality metrics are used as indicators of patients’ impairment level [40–42], it is crucial that the technological rehabilitation solution minimizes its own impact on these metrics. Our study is a first step in that direction as we showed that HMDs allow participants to train movements with better quality, reducing the negative impact of the current technological solution using 2D screens on movement execution. Nevertheless, we note that in our experiments we did not compare the movements performed with either of the visualization technologies with reaching movements in the real world, as this has already been evaluated in previous literature (e.g., [19, 21, 22]).

### HMDs might lower the cognitive load

We expected to observe a lower cognitive load with the HMDs compared to the 2D screen. However, we did not observe significant differences in the accuracy of the counting task between visualization technologies in the elderly participants. Similarly, the preliminary results from the five brain-injured patients do not indicate any potential effect of the visualization technology on the cognitive load measured with the counting task. Although we expected the elderly and brain-injured populations to be more sensitive to a possible change in cognitive load—as old age is associated with cognitive decline [43] and patients might suffer from cognitive impairment [3]—our results are in line with our previous experiment with healthy young participants [23].

We observed that the counting task accuracy was relatively high in both elderly and brain-injured populations, indicating that the parallel cognitive task might have been too easy to elicit enough mistakes to see a difference across the visualization technologies. We reduced the difficulty of the cognitive task from our previous study with healthy young participants by reducing the number of total fruits (120 in the previous experiment, 102 with elderly participants, and 48 with brain-injured patients) and the maximum number of fruits in a block (24 in the previous experiment, 18 with elderly participants, and 12 with brain-injured patients) to adapt to the new populations. However, it seems that we ended up with a not challenging enough parallel cognitive task. The use of other cognitive tasks such as continuous monitoring—e.g., measuring the reaction time to  simple stimuli, such as a color change [44]—, sensory discrimination—e.g., measuring the reaction time to recognize a given stimulus, such as a specific haptic signal [45]—, or arithmetic operations—e.g., backwards counting [46]—might be more challenging. Furthermore, the non-continuous nature of the cognitive and motor tasks in our experiments might have led to participants prioritizing the cognitive task over the motor task, resulting in a degradation of the movement quality metrics while the accuracy of the cognitive task remained high.

Regarding the self-reported cognitive load evaluated with the RTLX questionnaire, we found no significant difference across visualization technologies in the elderly participants, in line with our previous results with young healthy participants [24]. However, in the small group of brain-injured patients, the self-reported cognitive load appeared to be higher with the 2D screen than with AR, which also seemed higher than with IVR. This could potentially be due to the known cognitive impairments in brain-injured patients, who might have a different sensitivity to the potential cognitive load induced by the visuospatial transformation with the 2D screen. However, this observation must be further evaluated with a larger sample size of patients.

Importantly, we found significant differences across visualization technologies on the movement onset—i.e., the time lapsed between the appearance of the fruit and the start of the movement. Participants were asked to start saying aloud the counting value and the type of fruit before moving. Several studies have found an association between cognitive load and reaction times [45, 47]. We found that IVR significantly reduced the movement onset, compared to AR and 2D screen conditions in the elderly participants. Movements performed with AR also resulted in significantly shorter movement onset times than with the 2D screen. This difference was more obvious in movements that required moving towards fruit locations that involved the depth dimension. A similar trend was observed in brain-injured patients. Thus, performing reaching movements visualized on the 2D screen, especially those involving the depth dimension, might be associated with a higher cognitive load. This is consistent with previous literature on motion planning showing that when visual and proprioceptive feedback require recalibration, e.g., reaching in a visuomotor rotation environment [48], reaction times increase, likely due to the need to mentally transform the visual information to intrinsic coordinates for motion planning in 3D. This mental transformation may be especially demanding on the computer screen for targets in the depth dimension, causing prolonged reaction times. However, we cannot assume with certainty that longer movement onsets reflect higher cognitive load, as the onset was computed with a fixed velocity threshold. Thus, differences in pure motor aspects (i.e., the movement speed) might also lead to differences in movement onset. Yet, differences in the peak velocity between visualization technologies in the brain-injured patients were rather small, while the differences in the movement onset between these conditions were more obvious. Similarly, the differences in the peak velocity between the AR and 2D screen conditions were not significant in the elderly participants, while differences in the movement onset between these conditions reached significance.

The fact that participants could adapt their task performance strategy (i.e., take more time to count before reaching) may have mitigated the subjectively experienced cognitive load and, therefore, differences in the questionnaires across conditions could not be observed. Other cognitive tasks requiring continuous attention may be more powerful in detecting changes in the cognitive load, such as counting (backwards), performing simple arithmetic’s (for example, subtracting 7 starting from 100), citing the alphabet, etc.

To conclude, our results did not show differences between visualization technologies in the cognitive load, measured subjectively with the RTLX questionnaire and objectively with the cognitive counting task. However, we observed longer reaction times in the 2D screen condition, suggesting that the movement visualization on 2D screens might, indeed, increase the cognitive load. Importantly, the first self-reported assessments with brain-injured patients suggest a lower cognitive load when visualizing their movements with HMDs.

### HMDs do not significantly impact motivation

We expected that participants’ motivation would be higher with HMDs than with the 2D screen, either indirectly due to the improved movement quality or directly due to the more naturalistic movement visualization [24, 49]. However, contrary to our expectations, we did not find differences in participants’ motivation across visualization technologies. This result differs from the one reported in our previous experiment with young adults, where higher motivation was observed with IVR HMD compared to the 2D screen [24].

This could be interpreted as a potential reduction in the interest of elderly participants in new technologies. In our previous study, we recruited young adults from 19 to 42 years old. Other similar studies that found higher motivation when practicing with HMDs vs. 2D screens also included only young participants, e.g., in Born et al. participants were between 18 to 24 years old [50]. Similarly, in the study of Ijsselsteijn et al. [49], authors found that a higher immersion led to a higher motivation with a study population closer to our previous study (mean 41.3 years old). Thus, the high motivation associated with the use of HMDs might be age dependent. This difference highlights the importance of having studies with an age-matched population before drawing conclusions for clinical applications.

Nevertheless, it is important to note that, in the experiment with elderly participants, the recreated virtual environment was more complex than the one employed in our experiment with young participants, as it represented the real world with a higher fidelity. Being immersed in a simpler and less realistic virtual environment might potentially have increased the young participants’ motivation as they might have felt immersed in a virtual environment different than the real room. On the contrary, the realistic virtual environment employed with the elderly participants might have reduced their potential interest on IVR, as they were just immersed in a virtual environment that did not differ much from the real room.

### HMDs seem to enhance usability only in brain-injured patients

We also expected the more naturalistic movement visualization offered by HMDs to increase the system usability. However, the differences in usability between visualization technologies were not significant in the elderly participants, while they remain rather high through all the conditions. This contrasts with the first results observed with brain-injured patients, who rated the 2D screen visualization as less usable than HMDs. However, the between-subject variability in the usability scores of the 2D screen is rather large compared to the IVR HMD. More patients are needed to confirm this difference, but our preliminary results seem to point out that HMDs are perceived as more usable than 2D screens by brain-injured patients.

Yet, it remains unclear why the elderly participants did not rate the IVR HMD as more usable than the 2D screen, as observed in the younger population [24] and brain-injured patients. A potential rationale might be that elderly participants are less familiar with new technologies than young adults. Differences between elderly participants and brain-injured patients could arise from differences in the complexity of the whole system—one of the aspects rated in the system usability questionnaire [34]—between experiments. With the elderly participants, the overall system had a relatively low complexity as they were only holding a virtual controller in the 2D screen condition. Having to wear an HMD might be a significant addition in complexity, compensating for the more naturalistic visualization. However, in the experiment with brain-injured patients, the entire system setup included the mechanical exoskeleton attached to their paretic arm. The addition of a wearable display might not have been perceived as a significant increase in the complexity of the entire system. This suggests that the combination of HMDs with rehabilitation devices is technically feasible and well accepted by the clinical population. Yet, this should be further studied with a larger population, including not only patients, but also therapists.

To conclude, our results suggest that the addition of new technologies such as HMDs has no negative impact on the system usability, as elderly participants reported equally high usability (> 80/100) compared to a conventional 2D screen. The fact that the usability rating of our elderly participants did not differ between the likely highly familiar computer screen and novel HMDs is remarkable and underlines the acceptability of HMDs in elderly populations. Moreover, our first insights gained in brain-injured patients suggest that the clinical setting could especially benefit from the use of HMDs.

### Study limitations and future research

The most limiting aspect of our study is the small sample size of neurologic patients that prevented us from running statistical analyses and draw conclusions over the measured data. Unfortunately, the current COVID pandemic limited the access to the clinics. Continuing this study with more brain-injured patients, once restrictions are lifted, is our future goal. Yet, we believe that the insights gained in this feasibility study are important to the rehabilitation community.

A second limitation of our study is how we measured cognitive load. The measurement with a dual-task paradigm is assumed to be a more direct and objective measurement technique than questionnaires [51]. However, the validity of our counting task as a dual task might be compromised, as participants might have prioritized the cognitive task over the motor task as suggested by the observed differences in the movement onset. To avoid relying on subjective reports and to have a more direct measurement of cognitive load, future research should integrate physiological measures of cognitive load previously shown to be reliable, such as skin conductance [52], eye movements [53], pupil dilatation [54, 55], as well as heart rate variability [56], and electroencephalography (EEG) [57]. Similarly, other metrics could be used to assess movement quality. For example, less discrete correlates of movement smoothness than the number of velocity peaks, such as the jerk, are interesting metrics to complement future analyses [42].

Further, there were some technical differences across visualization modalities. The perceived contrast in AR displays depends on its luminance and the environment lighting conditions [58]. We dimmed our experimental room (Experiment 1 and 2) and had a black board placed behind the task workspace (Experiment 2) to remove background details and to ensure that the projected virtual elements would be easily perceived. However, it is still possible that the virtual environment/task was more difficult to perceive in the AR than the other display modalities, potentially affecting outcome metrics. For future experiments, environment lighting conditions should ideally be reported so that it can be ensured that the contrast of HMDs is similar between different testing environments.

Further, our displays differed in their focal depth, perceived resolution, and viewing distance, potentially influencing participants’ VR experience (e.g., how well they perceived the controller’s position). The focal depth describes the distance between the eye and the projected virtual plane. While for the AR HMD the focal depth is relatively small (i.e., distance from the eye to the transparent screen/glass), the focal depth of the IVR HMD approximately matches with the hand workspace. The screen presented the largest focal depth and is equal to the distance between participant’s eyes and screen. Further, the perceived resolution (pixel/inch) was also different across displays. In this regard, the IVR HMD is of relatively poor and the 2D screen of relatively high quality. Finally, the viewing distance, i.e., the visual size of the perceived virtual elements, was smaller for the 2D screen than the HMDs, due to the position of the screen with respect to the participant (approx. 80 cm). Overall, none of the three display types was equipped with fully ideal parameters, rather, each display type presented strengths and weaknesses.

Another limitation, which affects only the experiment with brain-injured patients, might be the potential visuohaptic conflict between the haptic stimuli—due to the weight support applied by the assistive device—and the visual absence of the device in the VE, i.e., the exoskeleton was not visible in the VE. Although it is unknown how this sensory conflict might have affected the patients in their movement quality or motivation and cognitive load reporting, recent evidence suggests that not visualizing assistive devices during training in immersive VR does not affect the users’ motivation, performance, nor visual attention, at least in a healthy young population [59].

Finally, our elderly population likely presented age-related vision deficits, i.e., the gradual loss of the eyes’ ability to focus on near objects (presbyopia) and gradual clouding of eye lenses leading to blurry vision (cataracts). Our clinical population, in contrast, included two younger participants that may not yet be affected by age-related vision deficits. These younger participants were possibly more prone to the so-called vergence-accommodation conflict associated with HMDs, i.e., the mismatch between the distance of the virtually rendered 3D object (vergence) and the focusing distance required for the eyes to focus on that object on the screen (accommodation). The vergence-accommodation conflict may have affected the depth perception of the virtual content and enhanced the visual fatigue in younger participants compared with the elderly, and, therefore, influenced our outcome metrics [60].

## Conclusion

This study presents results from two experiments performed with twenty elderly participants and a small group of five subacute brain-injured patients to evaluate and compare the effect of different visualization technologies (an HTC Vive Pro for immersive VR, a Meta 2 for augmented reality, and a computer 2D screen) on movement quality, cognitive load, motivation, and usability.

The more naturalistic movement visualization and increased depth perception with head-mounted displays improved the quality of the 3D reaching movements, compared to a conventional computer 2D screen. The HMDs might also have reduced the cognitive load, as measured by the time between stimulus presentation and movement onset. However, we did not find significant differences in subjective self-reports of cognitive load or in counting accuracy in the parallel counting task in the elderly healthy participants. Finally, although elderly and clinical populations might not be familiar with HMDs, participants rated them as highly usable, encouraging their usage in future VR-based rehabilitation interventions.

## Data Availability

The dataset presented in this study can be found online in the following repository: https://zenodo.org/record/6631771#.YqV_HXZBw2x.
